# Has Being Lost While High-Altitude Mountaineering Become Less Frequent? A Retrospective Analysis from the Swiss Alps

**DOI:** 10.3390/ijerph19031844

**Published:** 2022-02-06

**Authors:** Benedikt Andreas Gasser, Fabian Schwendinger

**Affiliations:** Division of Sports and Exercise Medicine, Department of Sport, Exercise and Health, University of Basel, 4052 Basel, Switzerland; fabian.schwendinger@unibas.ch

**Keywords:** map reading, cognitive skills, alpine environment

## Abstract

Background: High-altitude mountaineering is becoming more popular. Despite technical developments such as global positioning systems, mountaineers still lose their way. This study aimed to analyze characteristics of alpinists that lost their way while high-altitude mountaineering in Switzerland. Material and Methods: Data from the central registry of the Swiss Alpine Club between 2009 and 2020 were retrospectively analyzed. Changes in the number of cases and severity of injuries over time were examined using simple linear regression models. Descriptive analyses were performed for age, time of emergency occurrence, and factors associated with being lost. The Mann–Whitney U test assessed between-sex comparisons. Results: Of the 4596 emergency cases during the observation period, 275 cases (5.9%) were due to being lost (76.4% male). A mean of 22.9 ± 9.6 cases per year was detected. The number of cases did not change significantly over time. Similarly, this was the case for the NACA-Score (National Advisory Committee for Aeronautics Score) with the majority of mountaineers remaining uninjured (77.8%). The median age was 42 (35–54) years for the full sample and 45 (35–56) years and 40 (33–48) years for males and females, respectively. Fog or weather changes, exhaustion, and inadequate tour planning (time and darkness) were frequently documented by rescuers as perceived reasons for being lost. Regarding the time of emergency occurrence, three peaks were detected, around 10 am, 5 pm, and 8 pm. Conclusions: Our findings show that the number of emergencies due to being lost was stable during the 12-year period. Furthermore, we presented factors that might be associated with losing one’s way during mountaineering. These results may form an important basis for future studies determining risk factors for being lost and the prevention of such emergencies.

## 1. Introduction

Mountain sports activities have experienced increasing popularity, and increasing numbers of people are active in the mountains [1,2,3]. For high-altitude mountaineering, an annual number of 150,000 practitioners is estimated in Switzerland [4]. Apart from the well-known health benefits, the apparent risks for accidents and emergencies should not be neglected [1,2]. Falls, illnesses, lightning strikes, or becoming lost can potentially occur during these mountain sports activities [3,4,5,6].

Over the past decade, considerable progress has been made in the development of global positioning systems (GPSs), altimeters, and mobile reception. These allow the mountaineers to more easily locate their current position on the map and facilitate their arrival at a secure point, such as a cottage or a trail [7,8]. Mountaineering maps are constantly updated and of high quality, thus, facilitating tour planning. Recommendations by the Swiss Alpine Club (SAC) in combination with their maps and relatively easy adaptable concepts (performance km) allow for an adequate estimation of the duration and difficulty of a route [9,10,11,12].

Becoming lost during high-altitude mountaineering is an area of research that has received little attention. Data on the number of such emergencies over the last decade are of interest in terms of frequency and relevance. Furthermore, the characteristics of alpinists becoming lost are unknown. These data would, however, form an important basis to determine risk factors for this type of emergency. Characterizing victims of emergencies has proven to be valuable in other types of alpine emergencies, such as falls and cardiovascular events during mountain hiking as well as mountaineering [2,13,14,15].

Studies showed that sex might be associated with the occurrence of other types of mountain emergencies [2,13,14,15]. Moreover, there are indications for differences in safety behavior between men and women, with women being more risk averse than men [16]. For instance during mountain hiking, the sex ratio for non-fatal accidents was 55% female and 45% male; for fatal accidents, the female-to-male ratio was 28% and 72%, respectively [2,15]. Hence, whether sex also plays an role for being lost is of interest.

This study aimed to analyze whether the number of cases of being lost during high-altitude mountaineering has changed over the past 12 years in the Swiss Alps. Furthermore, we aimed to describe characteristics that may be associated with losing one’s way.

## 2. Materials and Methods

### 2.1. Study Design

This was a 12-year retrospective study (2009–2020) based on data of the central registry of the SAC, focusing on emergencies caused by persons becoming lost during high-altitude mountaineering in the Swiss Alps. The Ethics Committee of North-western and Central Switzerland granted ethical approval for the secondary data analysis.

### 2.2. Population

All mountain emergencies in the Swiss Alps requiring official rescue organizations to be mobilized are documented in the central registry of the SAC. The registry contains data from the Swiss Air Rescue Service, Air Glaciers Lauterbrunnen, Air Glaciers Sanenland, Register SAC, Kantonale Walliser Rettungs organisation, Snow and Avalanche Research Institute Davos, and the cantonal police registers. A first classification according to the cause of the emergency was made by these services, which was later checked by the study authors during the analyses. Mountain emergency is a definition for all events in which mountaineers receive the help of mountain rescue services or are affected by mountain hazards [9,10,17]. This also applies to illnesses and evacuations of uninjured mountaineers. Each mountain emergency included the emergency number used to make the call, date, rescue organization, event, place, canton (federal state in Switzerland), activity, NACA-Score (National Advisory Committee for Aeronautics Score; see Table 1), nationality, date of birth, sex, place of residence, coordinates, and a short report [18,19]. The short report contained information about the circumstances and perceived reasons of the emergency from the rescuers’ perspective. The term ‘blocked’ was defined as being no longer able to move forward or backward. Data were available for the period from 2009 to 2020. For the present study, all cases due to being lost were of interest.

### 2.3. Statistical Analyses

Data in text and tables are presented as median (IQR), mean ± SD, or absolute and relative frequencies. A normal distribution of the data was verified graphically using quantile–quantile plots. The direction of the alpinist at the time of the emergency was categorized as ‘descent’, ‘ascent’, or, if this information was not available from the short reports, as ‘unknown’. Similarly, the consequences of being lost were categorized as ‘blocked’, ‘losing general orientation’, or ‘unknown’ if this information could not be extracted from the short reports. To analyze potential differences between sexes, the Mann–Whitney U test was used. Changes in the number of cases and NACA-Score over the observation period were examined using simple linear regression models. Statistical significance for the two-sided tests was set to 0.05. All statistical analyses were performed in R version 4.0.3 [20].

## 3. Results

Out of a total of 4687 cases of emergencies during the observational period, 275 cases (5.9%) of alpinists that were lost were identified, of which 210 (76.4%) were male. The median age of those that became lost was 42 (35–54) years. Male alpinists were significantly older than females (median [IQR]: 45 (35–56) years vs. 40 (33–48) years; *p* < 0.01). Ninety-two (33.5%) were from Switzerland, 41 from Germany (14.9%), 18 from Italy (6.5%), 18 from Austria (6.5%), and 15 from France (5.5%). Furthermore, 16 were from the Netherlands (5.8%), 15 from Great Britain (5.8%), 11 from Czech Republic (4%), 11 from Poland (4%), 10 from Spain (3.6%), and 8 cases from Belgium (2.9%). The remaining individuals were from Denmark, India, Slovakia, Canada, Portugal, Japan, Hungary, the Soviet Union, and the USA (7.2%).

**Figure 1:**
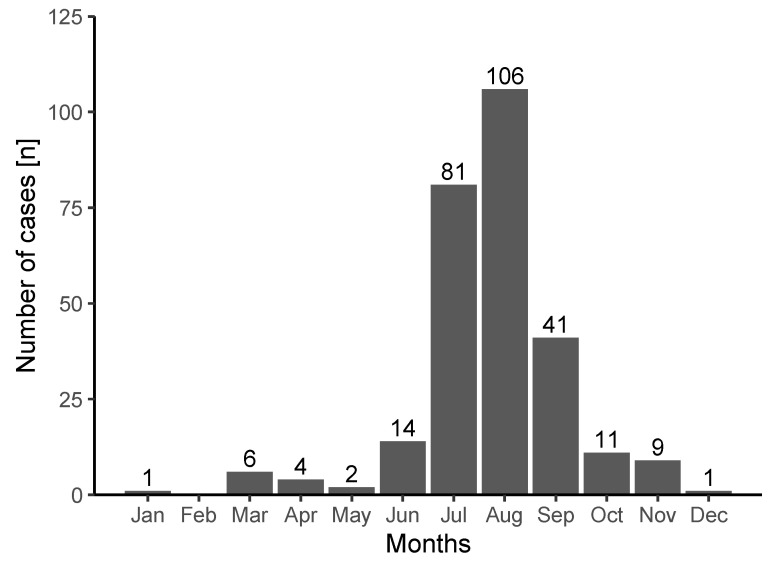
Number of cases due to being lost in the observational period (2009–2020) by month. Color coding: grey, male; black, female.

Figure 1 shows the distribution of cases during the observational period stratified by month, with an apparent peak during the two summer months of July and August. This distribution was similar for males and females.

On average, 22.9 ± 9.6 cases per year were detected in the full sample. When stratified by sex, 17.5 ± 7.3 cases per year were documented in males and 5.4 ± 3.4 cases per year in females. A simple linear regression of the full sample showed no significant change of the number of cases over the observational period, estimate (SE) = 0.4 (0.8) injuries per year; *p* = 0.652. Likewise, this was the case when analyzing males and females separately, estimate (SE) = −0.03 (0.6); *p* = 0.958 and estimate (SE) = 0.4 (0.3); *p* = 0.140, respectively.

The median NACA-Score was 0 (0–0) in both males and females (full sample 0 (0–0)), not significantly differing between sexes (*p* = 0.099). No fatal injuries were identified among the 257 cases. See Table 2 for more details on the distribution of the severity of injuries. The mean NACA-Score did not change over the observational period, estimate (SE) = −0.01 (0.02); *p* = 0.666. This was also the case for males and females, with estimate (SE) = −0.03 (0.03), *p* = 0.352, and estimate (SE) = 0.04 (0.03), *p* = 0.200, respectively.

**Figure 2:**
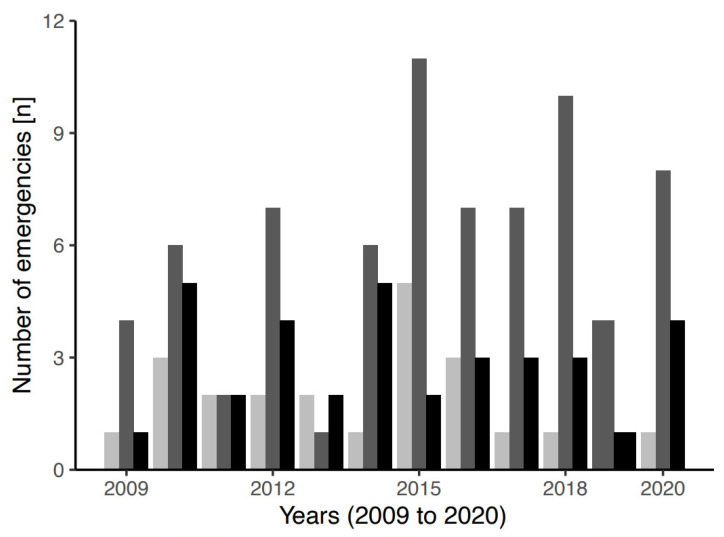
Number of emergencies due to alpinists being lost over the observational period (2009–2020) stratified by group size. Color coding: light grey, solo; grey, as a couple; black, in a group of three or more.

Figure 2 illustrates the group size at the time of the emergency. Of mountaineers who lost their way, there were a greater number in the category of couple compared to the category of individual or group.

Perceived reasons by the rescuer for being lost are summarized in Table 3.

**Figure 3:**
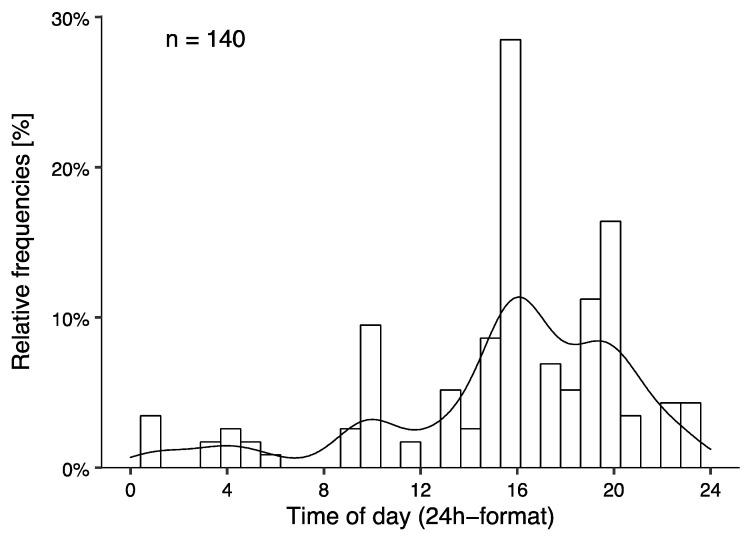
Histograms illustrating the time of the emergency.

Finally, a distribution of the time of the emergency is displayed in Figure 3.

## 4. Discussion

The major findings of the present study are that around two-thirds of the victims were from countries associated with the alpine region, the majority of alpinists that were lost were male (76.4%) and around 45 years of age, injuries were quite rare, and the number of cases was stable over the past 12 years, whereby environmental factors (i.e., fog and weather changes) and descending from the mountain seem to be associated with losing one’s way. Furthermore, emergencies peaked in the morning, afternoon as well as in the evening (Figure 3). The proportion of emergencies due to being lost in the high mountains was relatively low, considering that they only accounted for 5.9% of all mountaineering emergencies in the observational period in Switzerland. Around 45% of all mountain emergencies can be attributed to falls in the same data set (data not shown). Nevertheless, the objective should be to reduce the number of emergencies to a greater extent.

Considering that high-altitude mountaineering is mainly practiced in summer [21], it is not surprising that around two-thirds of all cases occurred in July and August (Figure 1) [21]. In addition to the high number of active alpinists in these months, meteorological aspects may play a role. Mean precipitation was previously shown to peak in July and August in the Swiss Alps [21,22]. This corresponds to the data from the emergency reports with 20.6% of the victims mentioning fog and 18.3% weather change. Thus, meteorological aspects may contribute to the majority of cases seen in summer and early autumn [22].

The number of alpinists that lost their way remained stable between 2009 and 2020. In addition, the number of SAC members during the respective time, which may be used as a proxy of mountaineering activity, increased by around 4% per year [3,4,6]. This indicates that the number of cases may have even decreased. A potential explanation could be the technological progress with global positioning systems, altimeters, meteorological services as well as cell phone reception which improve orientation on the mountains.

Likewise, the severity of injuries did not change over the observational period. The median NACA-Score was 0, indicating that cases of being lost are usually not accompanied by injuries. Accordingly, one could argue that such emergencies only yield a considerable economic burden. Searching for a missing person in the mountains can lead to rescue actions lasting for hours or even days, whereby rescue organizations need to rely on helicopters and actions can even be dangerous for the rescuers themselves. Thus, despite the low percentage of emergencies due to being lost, it seems important to minimize their occurrence.

Most alpinists who lost their way while mountaineering were a couple of two, followed by groups of three or more, and seldom a person mountaineering alone. Yet, it is not possible to imply causation from the available results. Nevertheless, the main causes of losing one’s way may in the core is a lack of map-reading skills and simply failing to gather or interpret weather information [9,10,17,23,24,25,26]. Thus, being accompanied by at least one person with sufficient map reading skills would potentially be enough to prevent becoming lost. However, map reading is a special skill that needs to be constantly practiced in order to have a sufficient skill level [23,24,25,26]. Thus, being skilled at map reading may be more important than group size. A relevant factor that may have biased this finding is experience. Solo mountaineers could be generally more experienced than others [23,24,25,26].

Information on the circumstances of the emergencies could form a basis for the development of prevention strategies and act as a starting point for future research. Being blocked was more frequently reported in females (41.9% vs. 27.7%) and a general loss of orientation more often in males (45.1% vs. 35.5%). Both situations may be avoided primarily by adequate tour planning and map reading. Additionally, for being blocked, technical skills (i.e., climbing skills) may come into play.

Environmental factors (i.e., fog and weather change) seem to be central elements associated with being lost. Thus, we recommend inquiring about current weather information prior to every trip. Moreover, it is advisable to keep an eye on the weather during the tour and act in a timely manner.

The descent was more often identified as the situation in which alpinists lost their way than the ascent. In a study on fall-related emergencies in mountain hikers, Faulhaber et al. [15] suggested that general fatigue may be a contributing factor. This may also be the case for losing one’s way, since 8.6% of cases reported exhaustion, even though the underlying mechanisms may differ. The close relationship between exhaustion/fatigue and cognition may affect map reading, predisposing alpinists to losing their way [23,24,25,26]. Nonetheless, this needs to be clarified in the future.

Chronometric data for this type of emergency during mountaineering are currently lacking. Studies on rock-climbing injuries reported that injuries most frequently occurred between noon and the early afternoon, i.e., 5 p.m. [27,28]. This is partly in line with the present data. Yet, there were additional peaks in cases in the morning around 10 a.m. and in the evening around 8 p.m. Since exhaustion seems to be an unlikely explanation, environmental factors, especially fog, seem more reasonable. Furthermore, underestimating the planned tour in terms of duration and difficulty may play a role by leading to time pressure and increasing darkness. This is supported by the data of the emergency reports but needs to be confirmed by future research that directly surveys the victims.

This study had several limitations. Firstly, the total number of active mountaineers in the Swiss Alps is unknown and difficult to estimate. The number of emergencies can therefore only be provided in absolute values. Applicable estimates of the number of active mountaineers in Switzerland would be important for future research. Secondly, as previously mentioned, studies using data from official emergency registries usually do not include emergencies that do not involve professional rescue organizations [2]. Thus, the actual number of emergencies was likely underestimated. Thirdly, the emergency reports presented did not contain standardized information. The utility thus varies greatly between cases. We recommend extending the routinely collected data by information about mountaineering experience, physical fitness, and general health status. Surveying the victims that lost their way would further enhance the quality of data. Fourthly, the factor of ‘experience’ might be highly relevant in the context of being lost. Knowing the terrain, being skilled at map reading, and being aware of potential weather changes might be beneficial for preventing being lost.

## 5. Conclusions

Based on the present data, we conclude that being lost in the Swiss Alps is relatively rare compared to other causes of mountain emergencies. The number of emergencies due to being lost has remained low and stable within the past 12 years despite potentially more alpinists being active over the observational period. This might be due to the technological advancement of electronic devices making orienteering easier. Alpinists are most of the time uninjured, but being lost is nonetheless associated with potentially long and expensive rescue operations. In particular, meteorological aspects (i.e., fog and weather change) and inadequate tour planning might be associated with losing one’s way. The findings of the present study may form an important basis for future studies determining risk factors for losing one’s way and the prevention of such emergencies.

## Figures and Tables

**Table 1:** Description of NACA-Score (National Advisory Committee for Aeronautics Score) [18,19].

NACA 0	No injury or disease.
NACA I	Minor disturbance. No medical intervention is required (e.g., slight abrasion).
NACA II	Slight to moderate disturbance. Outpatient medical investigation but usually no emergency medical measures necessary (e.g., fracture of a finger bone, moderate cuts, dehydration).
NACA III	Moderate to severe but not life-threatening disorder. Stationary treatment required, often emergency medical measures on the site (e.g., femur fracture, milder stroke, smoke inhalation).
NACA IV	Serious incident where rapid development into a life-threatening condition cannot be excluded. In the majority of cases, emergency medical care is required (e.g., vertebral injury with neurological deficit, severe asthma attack, drug poisoning).
NACA V	Acute danger (e.g., third grade skull or brain trauma or severe heart attack).
NACA VI	Respiratory and or cardiac arrest.
NACA VII	Death.

**Table 2:** Distribution of injury severity quantified by NACA-Score in the full sample and by sex.

NACA-Score	Total (*n* = 275)	Males (*n* = 210)	Females (*n* = 65)
0—no injury	214 (77.8)	165 (78.6)	49 (75.4)
I	47 (17.1)	35 (16.7)	12 (18.5)
II	8 (2.9)	5 (2.4)	3 (4.6)
III	5 (1.8)	4 (1.9)	1 (1.5)
IV	1 (0.4)	1 (0.5)	0 (0)
V to VII—death	0 (0)	0 (0)	0 (0)

Note: Data are presented as absolute frequencies with relative frequencies in brackets. A higher NACA-Score indicates a more severe injury. Abbreviations: NACA-Score. National Advisory Committee for Aeronautics Score.

**Table 3:** Perceived reasons and associated factors with being lost that were extracted from emergency reports stratified by sex. The topic is in italic.

Variables	Total (*n* = 257)	Males (*n* = 195)	Females (*n* = 62)
*Environmental factors* ^†^			
Fog	53 (20.6)	42 (21.5)	11 (17.7)
Weather change	47 (18.3)	38 (19.5)	9 (14.5)
*Tour planning* ^†^			
Darkness	17 (6.6)	14 (7.2)	3 (4.8)
Time	23 (8.9)	17 (8.7)	6 (9.7)
Pathless terrain	17 (6.6)	11 (5.6)	6 (9.7)
*Direction*			
Descent	61 (23.7)	50 (25.6)	11 (11.7)
Ascent	25 (9.7)	18 (9.2)	7 (11.3)
Unknown	171 (66.5)	127 (65.1)	44 (71.0)
*Subjective reasons* ^†^			
Exhaustion	22 (8.6)	18 (9.2)	4 (6.4)
Anxiety	2 (0.8)	2 (1.0)	0 (0.0)
*Consequences*			
Blocked	80 (31.1)	54 (27.7)	26 (41.9)
Loosing general orientation	110 (42.8)	88 (45.1)	22 (35.5)
Unknown	67 (26.1)	53 (27.2)	14 (22.6)

Note: Data are presented as absolute frequencies with relative frequencies in brackets. **^†^** Multiple answers were possible. The term ‘blocked’ implies being no longer able to move forward or backward.

## Data Availability

For legal reasons data is not accessible.
